# Computational Perspectives on Cognition in Anorexia Nervosa: A Systematic Review

**DOI:** 10.5334/cpsy.128

**Published:** 2025-04-07

**Authors:** Marta Radzikowska, Alexandra C. Pike, Sam Hall-McMaster

**Affiliations:** 1 Max Planck Institute for Human Development, Berlin, Germany; 2 Department of Brain and Cognition, University of Amsterdam, Netherlands; 3 Department of Psychology and York Biomedical Research Institute, University of York, United Kingdom; 4 Department of Psychology, Harvard University, United States of America

**Keywords:** anorexia nervosa, eating disorders, computational modelling, reinforcement learning, decision-making, cognitive flexibility

## Abstract

Anorexia nervosa (AN) is a severe eating disorder, marked by persistent changes in behaviour, cognition and neural activity that result in insufficient body weight. Recently, there has been a growing interest in using computational approaches to understand the cognitive mechanisms that underlie AN symptoms, such as persistent weight loss behaviours, rigid rules around food and preoccupation with body size. Our aim was to systematically review progress in this emerging field. Based on articles selected using systematic and reproducible criteria, we identified five current themes in the computational study of AN: 1) reinforcement learning; 2) value-based decision-making; 3) goal-directed and habitual control over behaviour; 4) cognitive flexibility; and 5) theory-based accounts. In addition to describing and appraising the insights from each of these areas, we highlight methodological considerations for the field and outline promising future directions to establish the clinical relevance of (neuro)computational changes in AN.

## Introduction

Anorexia nervosa (AN) is an eating disorder (ED) characterised by severe restriction of energy intake relative to individual needs, persistent pursuit of weight loss efforts, and a preoccupation with low body weight (World Health Organization, 2022). It is estimated that, globally, up to 2% of women and up to 0.3% of men suffer from AN in their lifetime, with the mortality risk for AN estimated to be five times higher than the general population (Eeden et al., 2021). In conjunction with disordered eating, individuals with AN often experience psychological distress and a range of physiological issues such as cardiovascular dysfunction, electrolyte imbalances or amenorrhea (American Psychiatric Association, 2013). AN can be treated successfully, for example by using psychological interventions (Monteleone et al., 2022); however, in many cases, a major goal of treatment (particularly in hospital settings) is weight restoration (Lebow et al., 2017). Notably, even after successful weight restoration, individuals with an AN diagnosis often continue to make restrictive eating choices (Steinglass & Walsh, 2016), experience anxiety around food and body image (Steinglass, Albano et al., 2012), score highly on generalised anxiety rating scales (Kezelman et al., 2015) and have high rates of rehospitalisation or relapse (Khalsa et al., 2017). Alongside and perhaps explaining the focus on weight restoration, another factor contributing to the low success rates of available treatments is insufficient insight into the mechanisms that give rise to and promote persistence of AN symptoms. For example, the persistent preference for low fat foods seen in AN (Foerde et al. 2015; 2021) could stem from a variety of underlying cognitive changes, such as a relative increase in habitual control over behaviour (Foerde et al., 2021; Onysk & Seriès, 2022) or heightened self-control (King et al., 2016; Steinglass et al., 2012).

To make sense of behavioural changes seen in those with severely restricted food intake and persistent weight loss efforts, there is a growing interest in research examining the neurocognitive processes behind AN (Miles et al., 2020; Steinglass et al., 2019). This has revealed that AN is associated with impairments in cognitive control and decision-making (Smith et al., 2018), including reduced cognitive flexibility (Westwood et al., 2016; Wu et al., 2014) and poorer decision-making performance in situations with probabilistic outcomes (Guillaume et al., 2015). Research along these lines has provided a strong foundation for describing the kinds of cognitive changes that occur in AN. Nonetheless, the use of overt behavioural measures and traditional summary statistics can be limited when it comes to examining the latent mechanisms that give rise to these changes, including differences in task performance and AN symptoms.

A novel framework to address this gap and advance understanding of cognitive mechanisms that underpin maladaptive behaviour in AN comes from computational psychiatry. Computational psychiatry applies methodological and analytical tools grounded in mathematical models to study phenomena related to mental health disorders (Huys et al., 2016). By formalising hypotheses in mathematical terms, computational psychiatry often aims to measure latent mental processes in experimental settings, and test how such processes are related to neural activity, real-world behaviour and clinical symptoms (Adams et al., 2016; Huys et al., 2021). Computational work on psychiatric conditions such as depression (Huys et al., 2015), obsessive-compulsive disorder (Maia & McClelland, 2012) and schizophrenia (Adams et al., 2013) has demonstrated the promise of this approach for informing more comprehensive accounts of mental health conditions, better diagnostic criteria, and new treatments.

Recent years have seen a surge in interest in computational psychiatry approaches to the study of AN: here, we systematically review studies that have investigated cognition in AN using a computational framework. Our review aims to summarise central insights from this nascent field. A total of 20 articles were identified for final review using systematic search and inclusion criteria. The experimental methods, modelling paradigms, and results across studies were used to ascertain current themes in this new and exciting line of AN research. The five main themes identified in the field were: 1) reinforcement learning, 2) value-based decision-making, 3) model-based and model-free contributions to behavioural control, 4) cognitive flexibility, and 5) theory-based accounts.

## Methods

The methodology for this review was informed by The Preferred Reporting Items for Systematic reviews and Meta-Analyses (PRISMA) statement (Page et al., 2021) and previous systematic reviews in the field of computational psychiatry (Pike & Robinson, 2022).

### Search Strategy and Article Selection

**Figure 1:**
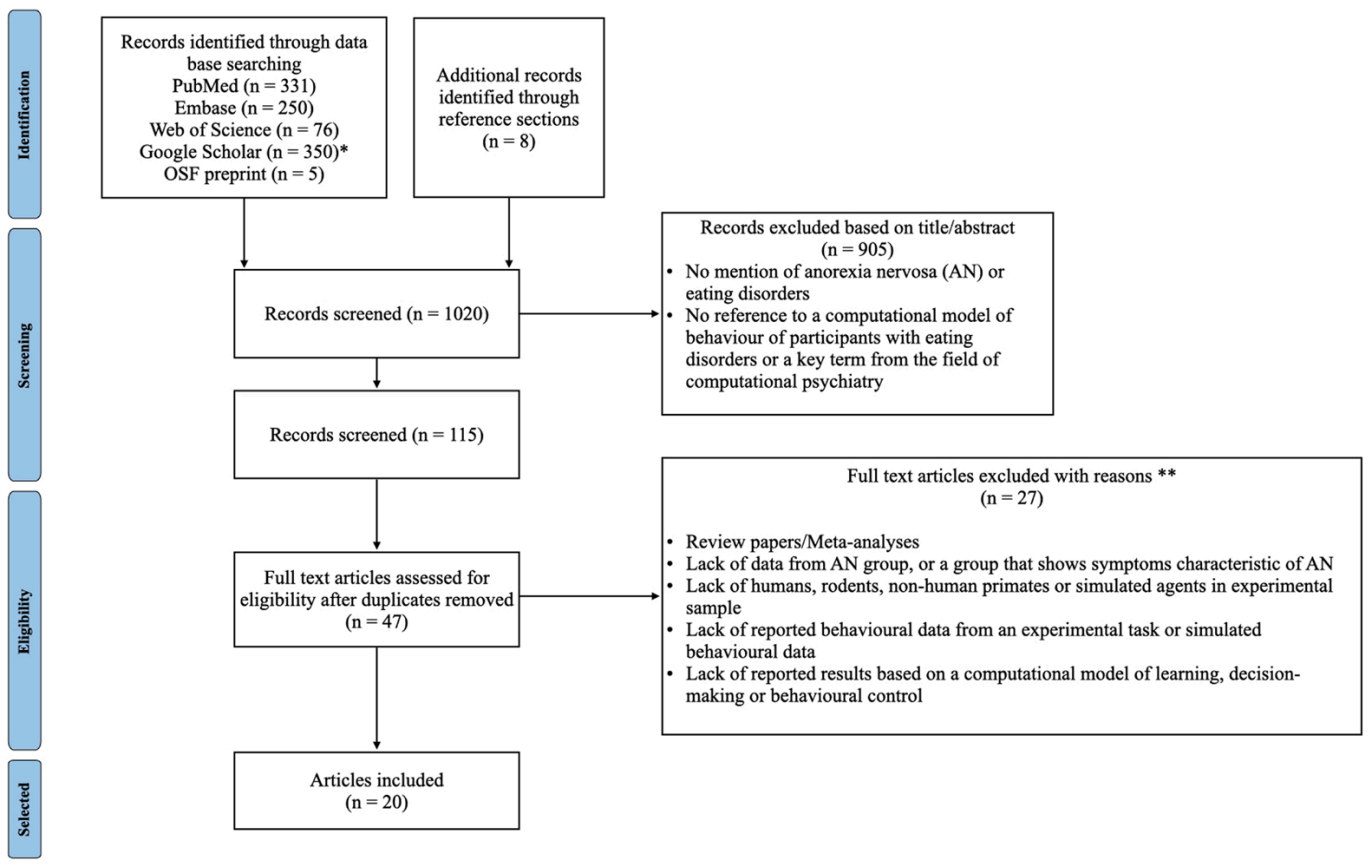
PRISMA flow diagram for papers included in this study. *Due to the large volume of results, the first 350 items, ordered by relevance, were screened for Google Scholar. **Theoretical papers that proposed a computational approach to AN but did not meet the experimental sample requirements were nevertheless included.

To identify relevant articles, PubMed and Embase were queried between 6/10/2022 and 20/10/2022, and Web of Science and Google Scholar were queried on 11/01/2024. This combination of databases was selected for high recall of relevant literature. Past research indicates that combining results from MEDLINE, Embase, Web of Science and Google Scholar has the highest overall recall in systematic reviews (Bramer et al., 2017). For the present review, PubMed was used rather than MEDLINE because it provides access to both MEDLINE and other sources. The search terms entered into these databases were ‘(‘*anorexia’ OR ‘eating disorder*’*)* AND (‘*computational psychiatry’ OR ‘computational model’)’*. Due to the high number of articles that Google Scholar returns for most searches, it is common to set an upper limit for how many results to screen (Bramer et al., 2017; Pike & Robinson, 2022). We set this limit at 350 papers. To identify relevant preprints, OSF Preprints was queried using keywords: ‘*anorexia’* AND (‘*computational psychiatry’ OR ‘computational model’)*. The selection process is summarised in Figure 1. Following identification, articles and preprints were screened based on their title and abstract.

Articles were included if their title/abstract:

1. Mentioned anorexia nervosa or eating disorders.
2. Referred to a computational model of behaviour, or included one of the following terms from computational neuroscience: prediction error, reinforcement learning, active inference, learning rate, learning curve, Bayesian inference, temporal discounting, model-free learning, model-based learning, exploration, or exploitation. Here and for subsequent selection criteria, a computational model was defined as a mathematical representation of a cognitive or neural process that included one or more latent variables.

After removing duplicates, papers were selected for full-text evaluation. During the evaluation stage, we excluded review papers and meta-analyses. Published articles and preprints were included in the systematic review if they met the following criteria:

1. Reported data from an AN group, or a group that shows symptoms characteristic of AN (e.g. restrictive eating, body image preoccupation). Eligible groups could consist of people who met a clinical threshold for AN, people who had recovered and/or were weight-restored, and people with subclinical symptoms. The motivation for including experiments with subclinical groups in the present review was that many behaviours associated with AN operate on a continuum (Maguire et al., 2008). Therefore, understanding computational profiles across severity levels provides insight into potential antecedents and risk factors for AN. We are careful to state specific samples used when discussing studies throughout the review.
2. Reported data from human participants, rodents, non-human primates or simulated agents as the experimental sample.
3. Reported behavioural data from an experimental task or simulated data.
4. Reported results based on a computational model of learning, decision-making or behavioural control (e.g. a reinforcement learning model).

Theoretical papers (n = 2) that proposed a computational framework of behaviour in AN were included even if they did not meet criteria 1–4. Thirteen papers met the above criteria and were selected. Eight additional sources were identified based on references from the 13 selected papers. The number of additional sources was relatively high because our initial search was not optimised to find articles focused on delay discounting. Seven of the additional sources passed the screening and evaluation criteria. References from these sources were further checked, but no additional papers were identified. This resulted in a final set of 20 papers.

### Data Extraction and Synthesis

We developed a checklist to extract key characteristics from each paper. It included: the aim of the research, hypotheses, study design, sample characteristics, task, computational framework (e.g. reinforcement learning, delay discounting), behavioural results, computational modelling results and the authors’ conclusions. In many cases, neuroimaging results were reported alongside behavioural data. However, since models of neural activity fall outside of the scope of the present review, we do not extensively cover these findings in the synthesis. Information from the checklist was formulated into themes based on the behavioural process, experimental task and models used to analyse results. A summary description of articles selected for systematic review is available in Table 1. The review itself is organised around five major themes: reinforcement learning, value-based decision-making, model-based/model-free control over behaviour, cognitive flexibility, and theory-based accounts. While presented as separate sections for clarity, these themes are concerned with interconnected cognitive processes. As such, some papers in the review appear in multiple sections (see Table 1). In each section, we first provide a brief background and describe important computational parameters, and later present the findings from studies included in the systematic review.

**Table 1:** Table summarising the characteristics of reviewed studies.


STUDY	THEME(S)	PARTICIPANT GROUPS WRITTEN AS GROUP NAME (SAMPLE SIZE): MEAN AGE (STANDARD DEVIATION), AND AGE RANGE, IF REPORTED	DIAGNOSTIC CRITERIA FOR THE AN GROUP(S)	COGNITIVE PROCESS	PARADIGM	KEY FINDINGS	CORRELATION OF MODEL PARAMETERS OR TASK PERFORMANCE WITH CLINICALLY RELEVANT FEATURES (SIGNIFICANT AT P < 0.05)

Bernardoni et al., 2018	Reinforcement learning	Acute AN (n = 36): 16.0 (2.6), 12–23 years HC (n = 36): 16.3 (2.6), 12–24 years	Acute AN: participated in a treatment programme	Learning from feedback, decision-making under uncertainty	Probabilistic reversal learning task	Increased learning rates after punishment in AN vs HC.	Not found

Bernardoni et al., 2021	Reinforcement learning	rec-AN (n = 34): 22.3 (2.8), 15–28 years HC (n = 63): 22.0 (2.9), 15–28 years	rec-AN: previously met DSM-IV criteria; no reported symptoms for > 9 months	Learning from feedback, decision-making under uncertainty	Probabilistic reversal learning task	Greater difference in learning rates between punished and rewarded trials in rec-AN vs HC. AN characterised by a learning style associated with low mood.	Not found

DeGuzman et al., 2017	Reinforcement learning	AN (n = 21): 15.2 (2.4), 13–20 years HC (n = 21): 16.4 (1.9), 11–20 years	AN: recruited from a treatment programme; adult participants met DSM-5 criteria Timepoint 1: Acute-AN Timepoint 2: AN-WR: after weight restoration to a BMI ≥ 19.5	Learning from feedback, punishment sensitivity	Monetary reward task	Negative PEs related to stronger responses in the caudate in Acute AN vs HC. Positive PEs related to stronger responses in the insula in Acute AN vs HC. At timepoint 2, no difference in PE signalling in AN-WR vs HC.	In AN, higher PE signalling in the caudate was associated with worse treatment outcomes.

Shott et al., 2012	Reinforcement learning	AN (n = 21): 25.2 (6.4) Subtype breakdown: AN-R (n = 11), AN-BP (n = 10) HC (n = 19): 27.3 (5.3)	AN: met DSM-IV criteria; recruited from a treatment programme	Feedback learning, decision-making	Category learning task	Impaired implicit category learning in AN vs HC.	Impaired category learning was associated with lower self-reported sensitivity to punishment and higher novelty seeking.

Filoteo et al., 2014	Reinforcement learning; cognitive flexibility	AN-WR (n = 19): 29.7 (6.6), HC (n = 35): 27.7 (5.1)	AN-WR: previously met DSM-IV criteria, no reported symptoms for > 12 months	Learning from feedback, set shifting	Category learning task with rule change	Increased learning speed during initial rule acquisition in AN-WR vs HC. Deficits in set shifting in AN-WR vs HCs.	In AN-WR, a more abnormal learning speed was correlated with shorter duration of weight restoration, and smaller change between the lowest registered and current BMI.

Wierenga et al., 2021	Reinforcement learning	AN (n = 42): 22.8 (9.6), 16–60 years HC (n = 38): 21.6 (4.3), 15–32 years	AN: met DSM-5 criteria; recruited from a treatment programme	Learning from feedback, punishment sensitivity	Probabilistic associative learning task	Lower learning rates and impaired learning from feedback in AN vs HC.	In AN, the magnitude of negative PEs during trials with punishment was associated with worse treatment outcomes.

Chan et al., 2014	Value-based decision-making	AN (n = 94): 25.6 (8.5) BN (n = 63): 26.9 (10.8) HC (n = 67): 25.5 (6.7) Data were collected across three sites.	AN at two sites (n = 81/94): met DSM-IV criteria AN one site (n = 13/94): EDDS questionnaire (Stice et al., 2000)	Probabilistic decision-making	IGT	Impaired task performance in AN vs HC, characterised by lower memory parameter estimates, indicating greater reliance on most recent outcomes for decision-making. Decreased loss sensitivity in AN vs HC in two out of three AN samples.	In AN, the learning/memory parameter from a prospect-valence learning model was positively correlated with BMI.

Verharen et al., 2019	Value-based decision-making	Study 1: AN (n = 60): 27.3 (9.9) HC (n = 55): 24.5 (8.3) Study 2: AN (n = 216): 22.3 (7.3)	AN: met DSM-IV criteria; recruited from a treatment programme	Probabilistic decision-making	IGT	Reduced loss aversion parameter in AN vs HC (gains and losses have similar impact on behaviour in AN, contrary to HC where losses have bigger impact on future choices).	Not found

Jenkinson et al., 2023	Value-based decision-making	Clinical study: Acute AN (n = 31): 24.9 (8.7). AN-WR (n = 23): 26.1 (7.5) HC low disordered eating (n = 38): 22.9 (3.3) HC high disordered eating (n = 35): 22.5 (4.7) Non-clinical studies: Study 0 + Study 1: (n = 170): 32.3 (10.6) Study 2 (n = 315): 23.4 (6.6)	Acute AN: met DSM-5 criteria; participated in a treatment programme AN-WR: diagnosed by a clinician as no longer meeting DSM-5 criteria; no reported symptoms for > 12 months HC high and low disordered eating: BMI, EDE-Q (Fairburn & Beglin, 1994) restraint scores	Decision-making under uncertainty, risk aversion	BART with a body-size condition	Risk taking in Acute AN and AN-WR was modulated by values related to increasing/decreasing body size. Corresponding computational changes in risk aversion, but not loss aversion, were seen in AN-WR.	Not found

Decker et al., 2015	Value-based decision-making	Timepoint 1: Acute AN (n = 59): 25.0 (7.5) HC (n = 39): 24.7 (7.6) Timepoint 2: AN-WR (n = 43) HC (n = 31)	Acute AN: met DSM-5 criteria; participated in a treatment programme Timepoint 2, AN-WR: measurements after weight restoration to a BMI ≥ 19.5	Intertemporal decision-making, inhibitory control	Delay discounting task	Lower delay discounting rates in Acute AN vs. HC. The difference at timepoint 1 was driven by AN-R. At timepoint 2, no difference in delay discounting rates in AN-WR vs HC.	Not found

King et al., 2016	Value-based decision-making	AN (n = 34): 15.7 (2.5), 12-22 years HC (n = 34): 16.1 (2.4), 12-22 years	AN: met DSM-IV criteria; participated in a treatment programme	Intertemporal decision-making, inhibitory control	ICT	No difference in delay discounting rates in AN vs HC, faster decision speed in AN vs. HC. Decreased brain activation in lateral prefrontal and posterior parietal regions associated with decision-making in AN vs HC.	Not found

King et al., 2020	Value-based decision-making	AN-WR (n = 36): 22.2 (3.3), 17–27 years HC (n = 36): 21.2 (3.4), 17-27 years	AN-WR previously met DSM-IV criteria; no reported symptoms for > 12 months	Intertemporal decision-making, inhibitory control	ICT	No differences in behavioural or brain measures in AN-WR vs. HC.	Not found

Ritschel et al., 2015	Value-based decision-making	AN (n = 34): 15.3 (2.7) rec-AN (n = 33): 21.7 (3.1) HC (n = 54): 18.8 (4.4)	AN: met DSM-IV criteria; recruited from a treatment programme rec-AN: previously met DSM-IV criteria, no reported symptoms for > 6 months	Intertemporal decision-making, inhibitory control	ICT	No difference in delay discounting parameter (*k*) in AN vs HC.	Not found

Steinglass et al., 2012	Value-based decision-making	Acute AN (n = 36): 24.8 (6.4) HC (n = 28): 25.9 (6.7)	Acute AN: met DSM-IV criteria; participated in a treatment programme	Intertemporal decision-making, inhibitory control	Titration task	Decreased temporal discounting in Acute AN vs HC.	Not found

Steinglass et al., 2017	Value-based decision-making	HC (n = 75): 29.0 (7.6) AN (n = 27): 27.7 (7.5) OCD (n = 50): 29.2 (5.8) SAD (n = 44): 30.0 (4.0)	AN: met DSM-IV criteria; participated in a treatment programme (different stages)	Intertemporal decision-making, inhibitory control	Titration task; ICT	Decreased temporal discounting in AN vs HC. No significant difference in HC vs OCD and SAD.	Anxiety was associated with decreased temporal discounting in all groups.

Foerde et al., 2021	Model-based and model-free control	AN (n = 41): 27.1 (7.0) HC (n = 53): 25.6 (5.0)	AN: met the DSM-5 criteria; recruited from a treatment programme	Goal-directed behaviour, learning, decision-making	Two-step decision task with monetary and food-specific condition	Decreased model-based contribution to learning in AN vs HC in monetary and food conditions.	Not found

Onysk & Seriès, 2022	Model-based and model-free control	ED (n = 35): 30.6 (4.5), 18-38 years HC (n = 32): 26.4 (4.6), 18-38 years	ED: reported being on restrictive diet in an attempt to lose weight; scored ≥ 14 on disordered eating and body image preoccupation on the EAT-26 (Garner et al., 1982) and AAI (Veale et al., 2014)	Goal-directed behaviour, learning, decision-making, body image preoccupation	Two-step decision task with monetary and body image disturbance condition	Decreased model-based and model-free contributions to learning in ED vs HC, with a greater effect in the body image disturbance condition.	The difference between model-based learning in the neutral and body image disturbance condition correlated with self-reported scores on disordered eating and body image preoccupation questionnaires.

Pike et al., 2023	Cognitive flexibility; reinforcement learning	rec-AN (n = 25): 23.5 (3.8) Subclinical ED (n = 25): 23.8 (2.8) HC (n = 32): 25.0 (6.4)	rec-AN: self-reported former AN diagnosis from a healthcare professional; no reported symptoms for > 12 months; did not meet DSM-5 criteria for ED subclinical ED: scored ≥ 20 on the EAT- 26 (Garner et al., 1982).	Adaptive learning, set shifting	The volatility task	Elevated learning rate adjustments in response to volatility in rec-AN vs HC.	Not found

Neuser et al., 2020	Theory-based accounts; value-based decision-making			Punishment sensitivity, reward sensitivity	Bandit task simulations	Low and invariable reward sensitivity associated with lower calorie intake in model simulations.	

Rigoli & Martinelli, 2021	Theory-based accounts; value-based decision-making			Choice evaluation		High reference point as a proposed explanation for behavioural manifestations of AN.	


*Note*. Abbreviations: AN = Anorexia Nervosa; AN-R = Anorexia Nervosa Restricting Subtype; AN-BP = Anorexia Nervosa Binge-Purge Subtype; HC = Healthy Control; AN-WR = Weight Restored Anorexia Nervosa; BN = Bulimia Nervosa; ED = Eating Disorder; OCD = Obsessive-Compulsive Disorder; SAD = Social Anxiety Disorder; rec-AN = Recovered Anorexia Nervosa; IGT = Iowa Gambling Task; BART = Balloon Analogue Risk Task; ICT = Intertemporal Choice Task; PE = Prediction Error; BMI = Body Mass Index; DSM-IV/5 = Diagnostic and Statistical Manual of Mental Disorders, 4th/5th Edition; EDDS = Eating Disorder Diagnostic Scale; EAT-26 = Eating Attitudes Test; AAI = Appearance Anxiety Inventory; EDE-Q = Eating Disorders Examination Questionnaire.

## Reinforcement learning

### Background

Altered processing of reward and punishment in AN is well documented. Questionnaire studies have shown that individuals with AN tend to report higher punishment sensitivity, reward sensitivity and harm avoidance (Jappe et al., 2011; Jonker et al., 2022; Fassino et al., 2002; Frank, 2021). Cognitive testing has shown that adults with AN tend to learn less from feedback overall, an effect that persists after weight restoration and correlates with symptom severity (Foerde & Steinglass, 2017). With recent developments in computational neuroscience, it is now possible to further investigate reward and punishment processing in AN. The main computational approach used to this end is reinforcement learning (RL). RL focuses on how agents use a trial-and-error process to anticipate outcomes and take actions that maximise their reward (and minimise their punishment) in a given context (Niv, 2009). Most RL models assume that learning happens when a deviation from expectations occurs, generating a prediction error. This error signal is then combined with a learning rate parameter, to update expected values of outcomes.

### Systematic Review Studies

Given the evidence of altered feedback processing in AN (e.g. Bischoff-Grethe et al., 2013; Foerde & Steinglass, 2017; Frank, 2013), a number of recent computational studies have sought to examine how learning changes in response to reward and punishment feedback by analysing RL parameters, with a focus on learning rates and prediction errors. We will now examine the findings from these studies.

### Evidence for increased learning from punishment in AN

Three studies in the review suggest increased learning from negative feedback in AN (Bernardoni et al., 2018, 2021; Filoteo et al., 2014). Bernardoni et al. (2018) observed a significant, but modest, elevation in learning rates following negative outcomes (i.e. punishment) in adolescents with acute AN compared to healthy controls (HCs). This elevation occurred after punishment but not reward, hinting at a learning bias specific to negative feedback. In their sample of adults who had recovered from AN, punishment learning rates themselves were not significantly different from HCs. However, the difference in learning rate between punishment and rewards was larger for the recovered AN group, suggesting that people recovered from AN continue to show a stronger relative response to punishment (Bernardoni et al., 2021). Further evidence that women who recover from AN might continue to show preferential processing of negative reinforcers was seen in a study reporting faster learning during the initial rule acquisition stage in a category learning paradigm (Filoteo et al., 2014). Model simulations showed that increasing a negative feedback *sensitivity* parameter was able to capture this behaviour in weight-restored AN. Hypersensitivity to punishment was associated with shorter maintenance of weight restoration and a smaller change in body mass index (BMI) between the lowest registered BMI and current BMI, implying that punishment sensitivity might be associated with recovery status (Filoteo et al., 2014).

### Evidence for increased prediction error signals in AN

Rather than examining reward or punishment per se, DeGuzman et al. (2017) focused on prediction errors, the difference between the reward expected and the reward obtained. Neuroimaging results showed stronger responses to prediction errors in the caudate and insula, during acute AN, compared with HCs. For context, independent studies have implicated the caudate in goal-directed action selection (Grahn et al., 2008), and the insula in error awareness (Klein et al., 2013) and decision-making (Uddin et al., 2017). Unexpected omissions in reward, which produce negative prediction errors, were associated with stronger responses in the caudate. Unexpected rewards, which produce positive prediction errors, were associated with stronger responses in the insula. Heightened prediction error responses normalised following weight restoration. However, stronger prediction error signalling in the caudate during the acute phase of AN was associated with worse treatment outcomes. Putting these results in context, one possibility is that individuals with AN are more responsive to negative feedback (Bernardoni et al., 2018, 2021; Filoteo et al., 2014), but especially when it violates expectations (DeGuzman et al., 2017).

### Evidence for decreased learning in AN

In contrast to the studies reviewed so far, there is also evidence for decreased learning in AN compared to HCs (Shott et al., 2012; Wierenga et al., 2021). RL modelling analyses of behaviour during an associative learning task showed decreased learning rates for both positive and negative prediction errors in AN (Wierenga et al., 2021). Prediction error magnitudes did not differ between samples; however, negative prediction error magnitudes in AN were associated with worse treatment outcomes.

### No evidence for a difference in learning in AN

In addition to positive results reported above, we note that some computational studies have compared learning rates between AN and HC groups, but did not find significant differences (Foerde et al., 2021; Verharen et al., 2019).

### Exploration

A key parameter controlling how much people explore different options and exploit high value options in standard RL models is called the inverse temperature (*β*), which measures the extent to which choices are based on differences between learned values (Daw et al., 2006). The majority of the studies in this review did not find significant differences in values of the inverse temperature parameter between AN and HCs, which could suggest similar levels of off-policy or exploratory behaviour. One exception was Wierenga et al. (2021), who reported lower inverse temperatures in AN compared to HCs during a learning task, even after participants had learned associations between different stimuli and their outcomes. This could reflect increased exploration in the sample, but could also reflect greater noise in the decision process or difficulties focusing on the task (Eshel & Roiser, 2010).

## Value-based Decision-making

### Background

AN symptoms, such as restrictive food choices and resistance to treatment despite dangerously low weight, can be viewed as decision-making impairments (Giannunzio et al., 2018). This has led to a vast body of research exploring whether altered decision-making in AN is specific to food and body weight, or whether changes extend beyond these contexts. In laboratory settings, decision-making is often assessed using choice tasks involving monetary rewards, such as the Iowa Gambling Task (IGT) or Balloon Analogue Risk Task (BART). Classical studies indicate reduced decision-making performance and lower risk thresholds in AN (e.g. Adoue et al., 2015; Bodell et al., 2014; see Howard et al., 2020 for review). Altered evaluation of future rewards has also been studied in AN, as a potential mechanism that underpins forgoing immediate food rewards in pursuit of longer-term weight-loss goals (e.g. Decker et al., 2015; King et al., 2016).

### Systematic Review Studies

To date, computational studies on decision-making in AN have focused on probabilistic decisions, risk aversion and temporal discounting. We will now review the findings from each of these areas.

### Probabilistic Decisions and Risk Aversion

Two computational studies have examined AN choices in probabilistic settings using the IGT (Chan et al., 2014; Verharen et al., 2019). Both found that individuals with AN had reduced performance compared to HCs, but reached different conclusions about the underlying mechanism. Computational modelling by Verharen and colleagues (2019) indicated that individuals with AN were less loss-averse than HCs. As a result, AN participants experienced negative outcomes more often and earned less reward on the task. In contrast, computational modelling by Chan and colleagues (2014) found that the best explanation for lower IGT performance was a decrease in the extent to which participants based their decisions on past trials, indicating potential impairments in learning and memory rather than unusual reward preferences. The precise mechanisms of impaired probabilistic choice in AN therefore remain open.

The main insight from computational research on risk tolerance has been that individuals with AN are less willing to take risks to earn rewards in general (Jenkinson et al., 2023). However, this willingness can change depending on the specific decision context. When rewards were tied to making an onscreen character slimmer, participants with acute and weight-restored AN became more willing to proceed with the trial despite an increased risk of losing money. The direction of this effect reversed when rewards were linked to increasing body size. Computational modelling confirmed corresponding changes in risk aversion, not loss aversion, for participants with weight-restored AN. Based on these results, the authors propose that general risk aversion may be a state-independent factor predisposing people to AN, while the value placed on changing body size may modulate risk aversion during specific decisions (Jenkinson et al., 2023).

### Delay Discounting

Delay discounting (also called temporal discounting) aims to measure how well one can delay the receipt of reward. The discounting process is often modelled with a hyperbolic function (see Green & Myerson, 2004 for review), in which the main computational parameter is the discount rate, *k*. This parameter captures the reduction in subjective value that an option incurs with increasing delays to receive its corresponding reward. Higher *k* indicates a greater preference for lower but immediate rewards versus higher but delayed rewards. Several studies have investigated temporal discounting in AN, but findings have been mixed. Some studies report that individuals diagnosed with the restricting subtype (AN-R) show reduced temporal discounting of delayed monetary rewards compared to HCs (Decker et al., 2015; Steinglass et al., 2012, 2017), a finding that has not been observed in individuals with the binge-purge subtype (AN-BP) (Steinglass et al., 2012), obsessive-compulsive disorder (OCD) or social anxiety disorder (Steinglass et al., 2017). Temporal discount rates normalised after weight restoration (Decker et al., 2015), suggesting that altered discounting is an illness-specific state, rather than a trait. In contrast, other studies report no difference in temporal discount rates between acute, partially restored and recovered adolescent AN and HCs (King et al., 2016, 2020; Ritschel et al., 2015). One plausible reason for the inconsistency could be the age of participants tested. Two studies reporting no difference in temporal discounting rates (King et al., 2016; Ritschel et al., 2015) tested participants around 10 years younger than studies reporting reduced discounting in AN (Decker et al., 2015; Steinglass et al., 2012; Steinglass et al., 2017). These results suggest that reduced temporal discounting may be a characteristic of adults with acute AN-R, but not adolescents with AN.

## Model-based and Model-free Control Over Behaviour

### Background

A well-established theory of AN proposes that disordered eating emerges as a goal-directed behaviour, where an individual purposefully restricts food intake and finds the outcomes rewarding. Over time, these behaviours shift from goal-directed to habitual control. Once habitual, behaviours that restrict food and reduce body weight continue to occur irrespective of their outcomes, resulting in the rigid, persistent, and compulsive symptoms of AN despite adverse consequences (Steinglass & Walsh, 2006; Uniacke et al., 2018; Walsh, 2013). In the computational literature, goal-directed and habitual behaviour have been operationalised as model-based and model-free learning. Model-based processes depend on mental maps of contingencies in the environment that can be used to simulate possible outcomes without experiencing them in real life. In contrast, model-free processes depend on trial-and-error learning and decisions are based on associations between the stimuli and learned responses. The contribution of these two systems to behaviour is often assessed using a two-step decision task and modelled with a ‘hybrid model’, in which choices are predicted using a weighted combination of model-based and model-free processes (Daw et al., 2011).

### Systematic Review Studies

To date, two studies have used computational approaches to examine model-based and model-free learning in AN (Foerde et al., 2021; Onysk & Seriès, 2022). Foerde et al. (2021) found decreased model-based learning in AN, in both food-specific and monetary tasks, suggesting a domain-general shift in the balance between goal-directed and habitual control over behaviour. This effect persisted even after weight restoration, ruling out the potential influence of starvation. This reduction in model-based learning was replicated in a subclinical ED group tested online (Onysk & Seriès, 2022). They found that the effect was most pronounced in experimental blocks where monetary reward was paired with an icon that participants had selected as being similar to their own body shape. Model-based learning in these blocks, relative to neutral blocks, was able to successfully predict scores on a self-reported disordered eating and body image scale, but not OCD-like behaviour.

In contrast to reduced model-based learning observed in both studies, Foerde at al. (2021) found that model-free learning remained intact in acute AN. This finding differed from the subclinical ED sample in Onysk & Seriès (2022), who also showed reduced model-free learning. One possible explanation for the difference could be the samples used in the two studies. Foerde et al. (2021) were unambiguously testing individuals with acute AN, whereas Onysk & Seriès (2022) were testing individuals with high levels of concern about eating, independent of a formal diagnosis. The latter sample could include a range of ED categories, such as subclinical forms of AN, bulimia nervosa or binge eating disorder. It is therefore possible that the precise balance of goal-directed and habitual control is distinct in acute AN compared to other ED populations.

## Cognitive Flexibility

### Background

Rigid and often ritualistic eating behaviours, perfectionism, and the strong preference for familiarity over new experiences commonly observed in AN patients have been linked to impairments in cognitive flexibility (Holliday et al., 2005). Cognitive flexibility refers to the ability to adjust behaviour to changing contingencies in the environment, and it is often studied using paradigms that require relinquishing previously learned rules and adapting behaviour to new contexts (Dajani & Uddin, 2015). Individuals with AN often exhibit reduced performance in this domain, using the same choice strategy for significantly longer after a contingency change than HCs (Steinglass et al., 2006; Wu et al., 2014).

### Systematic Review Studies

To better understand impairments in cognitive flexibility, Filoteo et al. (2014) used a computational model that assumed set-shifting depends on a competition between an explicit hypothesis testing system and an implicit learning system, implemented in a hybrid neural network where learning was controlled by RL rules. The setup had three parameters with potential relevance to set-shifting: 1) a parameter that captured decisions to follow unusual rules, which the participant had rarely used before, 2) a parameter that captured the tendency to continue using one rule regardless of feedback (perseveration), 3) a parameter that captured sensitivity to negative feedback. Increasing the perseveration parameter and decreasing the unusual rule selection parameter successfully accounted for set-shifting difficulties in weight-restored AN.

In contrast to research indicating reduced cognitive flexibility in AN, Pike et al. (2023) found evidence for greater cognitive adjustment in response to changing task demands. Here cognitive flexibility was examined from a different angle, focusing on how learning is calibrated to environments with volatile or stable contingencies. Since rewards in volatile environments can be better predicted from more recent feedback, learning rates were expected to increase in volatile environments compared to stable ones. Hence, the main idea was that the change in learning rate between volatile and stable task environments could be used as a measure of cognitive flexibility. Women recovered from AN showed greater adjustment of learning rates between volatile and stable blocks compared to HCs – opposite to the authors’ hypotheses, and, if anything, indicative of greater rather than reduced cognitive flexibility. Learning rate adjustments were comparable between a subclinical ED group and HCs, and conventional stay/switch analyses of overt behaviour did not reveal any group differences in flexible processing.

## Theory-based Accounts

In addition to the empirical results above, two computational theories of cognitive change in AN have been developed. The Reference Dependent Model of AN (RDMA) proposes that altered evaluation processes underlie the symptoms of AN (Rigoli & Martinelli, 2021). The model is grounded in the idea that people develop internal, subjective values for different situations, based on the outcomes they experience. Within RDMA, the process of transforming outcomes into subjective values rests on a reference point parameter (*μ_k_*). The reference point is an expectation about how good a specific environment, *k*, should be. This influences the subjective values of outcomes compared to this reference point. Outcomes better than the reference are experienced as rewarding, while outcomes worse than the reference are experienced as punishing. The RDMA uses this framework to propose two main changes in AN. The first is a high reference point across a range of situations (i.e. a general increase in *μ_k_* relative to HCs). Only outcomes that exceed this unrealistically high reference point are experienced as positive, leading to perfectionism. The second proposed change is an increased sense of control over body shape in AN, due to large differences in subjective value between actions that reduce body weight and inaction.

The second computational theory identified in this review proposes that low and invariable sensitivity to food reward may explain the behavioural symptoms of AN (Neuser et al., 2020). Reward sensitivity in this theory is scaling learned option values at the point of decision-making (akin to an inverse temperature), and not affecting reward outcomes observed during feedback. The effect of conceptualising sensitivity in this way is that, when the reward sensitivity is low, food choices cease to reflect the aspects of food that are normally appetitive, such as calorie density. Crucially, this sensitivity is not just characterised as an average that is reduced compared to HCs, but also by its variability over time and different physiological states. This forms a major tenet of the theory, which asserts that changes in sensitivity to food rewards over time, for instance in states of hunger and satiety, are narrower in individuals with AN than for individual HCs. To express this in more formal terms, individuals with AN are theorized to have both a lower average sensitivity to food rewards and a smaller standard deviation in their sensitivity distribution. These two factors could account for reduced calorie intake in AN, as high calorie foods are not treated as more valuable than other foods, and rigid eating patterns in AN, since fluctuations in how rewarding food is across time are constrained within a more narrow distribution (Neuser et al., 2020).

## Discussion

In this review, we have sought to systematically review findings from research applying computational methods to study mechanisms behind persistent behavioural changes seen in AN. Based on 20 articles reviewed, we have identified and described emerging themes in this new field. These themes centre on the computational investigation of: 1) reinforcement learning in AN; 2) value-based decision-making; 3) model-based and model-free control over behaviour; 4) cognitive flexibility; and 5) theory-based accounts. Below we summarise the main findings from this review and discuss the need to more directly explore the relationship between altered computational mechanisms and clinically relevant factors.

### Summary of Key Results

Broadly, computational studies indicate deficits in cognitive processes that guide behaviour in AN. However, there is considerable variability in findings across research. Based on the current literature, there is mixed evidence for whether reinforcement learning is abnormal in AN, with some studies not finding significant alterations in learning rate (Foerde et al., 2021; Verharen et al., 2019), other studies showing decreased learning from feedback in general (Wierenga et al., 2021) and other studies showing heightened processing of negative feedback (Bernardoni et al., 2018, 2021; Filoteo et al., 2014).

Several studies in this review reported changes in decision-making in AN. One found choice performance was impaired in AN due to a greater reliance on recent outcomes (Chan et al., 2014), whereas another suggested decreased sensitivity to losses as the central mechanism (Verharen et al., 2019). Choices in AN are also more risk averse in general, but risk aversion is reduced when actions lead to illness-consistent outcomes, such as reduced body size (Jenkinson et al., 2023). Alongside these findings, research on delay discounting indicates an increased preference for delayed rather than immediate monetary rewards in adults with acute AN, particularly AN-R (Decker et al., 2015; Steinglass et al., 2012, 2017). Altered delay discounting is not seen after weight-restoration (Decker et al., 2015; King et al., 2020) or in adolescents with AN (King et al., 2016; Ritschel et al., 2015).

Computational studies on model-based and model-free control reported that individuals with either AN or subclinical EDs use goal-directed (model-based) strategies for behavioural control less than HCs (Foerde et al., 2021; Onysk & Seriès, 2022). During both acute AN and following weight restoration, reduced model-based control was seen in both monetary and food-specific contexts (Foerde et al., 2021).

Finally, computational work on cognitive flexibility in AN has found mixed results. Some research has shown greater cognitive rigidity in AN, exhibited in a low level of exploration and a tendency to continue using the same decision strategy (Filoteo et al., 2014). However, another study observed more flexible adjustment in learning rates in response to changing task demands in a group recovered from AN (Pike et al., 2023).

### Impact of Recovery and Clinical Relevance

Only a few studies in this review found that model-derived parameters could predict the severity of AN symptoms. For example, the effect of body image preoccupation on the strength of model-based control over behaviour was able to predict scores on self-reported eating disorder scales and ED membership (Onysk & Seriès, 2022). Moreover, worse treatment outcomes were associated with elevated sensitivity to punishment, negative prediction error magnitudes and stronger prediction error signalling in the caudate (DeGuzman et al., 2017; Filoteo et al., 2014; Wierenga et al., 2021). The limited number of studies linking computational changes to patient symptoms highlights that more research will be needed to identify computational changes with functional significance, which have the highest predictive validity and translational value as neurocomputational markers of AN (see Paulus et al., 2016).

Another aspect of clinical relevance is whether computational differences resolve or persist after treatment. Current evidence indicates that prediction error signalling and delay-discounting rates normalise after weight restoration (Decker et al., 2015; DeGuzman et al., 2017). However, increased learning from punishment (Bernardoni et al., 2018, 2021) and decreased model-based learning (Foerde et al., 2021) persist after weight-restoration and appear to be trait-like. Based on this dichotomy, future longitudinal studies could map changes in computational parameters throughout the development and maintenance of AN. This could be used to establish computational profiles of AN progression, identify latent factors that are malleable or resistant to change, and to devise different intervention strategies for different phases of illness.

### Assessing Transdiagnostic and Specific Symptoms of AN

A question which remains is whether differences between AN and HCs identified in computational studies are specific to AN. For example, increased learning rates in response to negative feedback observed in AN groups (Bernardoni et al., 2018, 2021; Filoteo et al., 2014) have also been reported in patients suffering from mood and anxiety disorders (Pike & Robinson, 2022). In patients with mood disorders, this may lead to the progression of negative affect and negativity bias, driven by updating beliefs and behaviour in response to negative outcomes too quickly. Mood and anxiety disorders commonly co-occur with AN (Swinbourne & Touyz, 2007), but whether negative feedback processing is comparable between these disorders is currently unclear. This could be explored in transdiagnostic studies. One approach to studying transdiagnostic populations is computational factor modelling (CFM), which is used to explore associations between changes in cognitive mechanisms and specific symptom dimensions, rather than diagnostic categories (Wise et al., 2023). A recent study using CFM identified that deficits in model-based planning were associated with symptoms of disordered eating, OCD and addiction, suggesting that model parameters related to goal-directed control correspond to individual differences in compulsivity irrespective of diagnosis (Gillan et al., 2016). Future studies in the field could therefore use CFM to investigate whether computational changes are specific to AN or present in multiple conditions.

### Assessing Domain-general and Context-specific Symptoms of AN

The studies described in this review suggest that several cognitive changes in AN are domain-general, detectable in neutral contexts with monetary (rather than disorder-relevant) outcomes. Nevertheless, several experiments in this review addressed context-specific alterations in AN by including disorder-relevant stimuli (Foerde et al., 2021; Jenkinson et al., 2023; Onysk & Seriès, 2022). Deficits in goal-directed control in AN appear to be generalised, occurring in both neutral and food-relevant situations, rather than being specific to food-related decision-making alone (Foerde et al., 2021). At the same time, research from a subclinical ED population suggests that deficits in goal-directed control might be amplified in a context where body image concerns are made salient (Onysk & Seriès, 2022). Taken at a general level, this amplification in an illness-specific context fits with research suggesting that propensity for risk-taking in AN increases, when choices are linked to reductions in body size (Jenkinson et al., 2023). These findings indicate that AN is subject to both domain-general cognitive changes and context-specific changes, and that these can be distinguished using experimental paradigms with both neutral and illness-relevant conditions.

### Different AN Subpopulations

There is much variability in how AN groups are defined across the literature. Some studies described in this review included participants who completed the testing session in the acute state of AN upon admission to a treatment program (e.g. Bernardoni et al., 2018, Jenkinson et al., 2023). Other studies considered participants who were undergoing treatment and were weight-restored at the time of testing (e.g. Filoteo et al., 2014, King et al., 2020), or individuals with a prior AN diagnosis who have since recovered (e.g. Pike et al., 2023). Age is another an important sampling factor given that AN typically begins during adolescence, a developmental period that includes changes in prediction error responses (Hauser et al., 2015), gradual increases in learning rate (Master et al., 2020) and the emergence of model-based decision strategies (Decker et al., 2016). Computational processes might therefore differ in different AN subpopulations. For example, studies testing adolescent AN have not observed altered discount rates (Ritschel et al., 2015; King et al., 2016), but studies testing adults with acute AN-R have (Decker et al., 2015; Steinglass et al., 2012; Steinglass et al., 2017). Systematic comparison of AN subpopulations, based on factors like age and illness duration, could help to clarify which cognitive changes are risk factors for the development of an eating disorder, result from the disorder itself (e.g. due to malnutrition), contribute to its maintenance or relapse, or persist as cognitive ‘scars’ after recovery.

### Computational Comparability

Mixed evidence for altered learning and decision-making processes in AN highlights the need for reliable experimental paradigms. Inconsistent findings across studies could be the result of methodological differences, arising from the fact that studies use a range of different tasks, models and methods for parameter estimation. One way to increase the comparability of findings as the field advances could involve the introduction of a shared set of models and reporting criteria, including the assumptions of the fitting method used (for example, whether it guarantees convergence to true values or relies on approximations like variational Bayes). This way, new findings could be compared against a common baseline. A second possible explanation for mixed findings relates to an assumption often held in computational studies, which is that computational parameters have similar interpretations in different contexts. However, values of computational parameters and their interpretation might vary across time (Hauser et al., 2015) and tasks (Eckstein et al., 2022). The accurate interpretation of group differences in parameter values is particularly consequential for studies with clinical populations, such as AN, in which the eventual goal is to inform avenues for treatment. To enable a more nuanced investigation of computational processes driving AN, future work could test how the same sets of individual and group-averaged parameters change across different tasks, to better understand how these parameters should be interpreted in light of various task demands.

### Limitations

This review used systematic search and inclusion criteria to identify computational psychiatry studies of AN. The first search step used the terms ‘(‘*anorexia’ OR ‘eating disorder*’*)* AND (‘*computational psychiatry’ OR ‘computational model’)* for PubMed, Embase, Web of Science and Google scholar, and the terms ‘*anorexia’* AND (‘*computational psychiatry’ OR ‘computational model’)* for OSF preprints. These initial search terms were deliberately broad to avoid presupposing or predefining specific models or tasks. While this approach was able to capture key papers from the field, we cannot guarantee it was fully exhaustive, as the terms above could be absent from the title or abstract of otherwise relevant studies. Including more specific search terms at the first step, such as ‘reinforcement learning’ or ‘delay discounting’, could ensure a more exhaustive procedure for future reviews on computational psychiatry studies in AN.

## Conclusion

In this systematic review, we have outlined the current landscape of computational psychiatry in the context of AN by describing recent efforts to integrate computational neuroscience with the study of cognition and behaviour in AN. These efforts fall into five major themes: 1) reinforcement learning; 2) value-based decision-making; 3) model-based and model-free control over behaviour; 4) cognitive flexibility; and 5) theory-based accounts. While computational changes in AN have been reported in all five areas, results across studies remain variable. Moreover, very few studies have found associations between computational changes and condition severity or recovery status. Developing robust models, with a focus on how computational changes are related to clinical measures, remains an important objective for the field to bridge computational insights and clinical practice.
